# Antarctic Cryptoendolithic Fungal Communities Are Highly Adapted and Dominated by Lecanoromycetes and Dothideomycetes

**DOI:** 10.3389/fmicb.2018.01392

**Published:** 2018-06-29

**Authors:** Claudia Coleine, Jason E. Stajich, Laura Zucconi, Silvano Onofri, Nuttapon Pombubpa, Eleonora Egidi, Ashley Franks, Pietro Buzzini, Laura Selbmann

**Affiliations:** ^1^Department of Ecological and Biological Sciences, University of Tuscia, Viterbo, Italy; ^2^Department of Microbiology and Plant Pathology, Institute for Integrative Genome Biology, University of California, Riverside, Riverside, CA, United States; ^3^Hawkesbury Institute for the Environment, Western Sydney University, Penrith, NSW, Australia; ^4^Department of Physiology, Anatomy and Microbiology, La Trobe University, Melbourne, VIC, Australia; ^5^Centre for Future Landscapes, La Trobe University, Melbourne, VIC, Australia; ^6^Department of Agricultural, Food and Environmental Sciences, Industrial Yeasts Collection DBVPG, University of Perugia, Perugia, Italy; ^7^Section of Mycology, Italian National Antarctic Museum (MNA), Genoa, Italy

**Keywords:** Antarctica, endolithic communities, extremophiles, fungi, ITS meta-barcoding

## Abstract

Endolithic growth is one of the most spectacular microbial adaptations to extreme environmental constraints and the predominant life-form in the ice-free areas of Continental Antarctica. Although Antarctic endolithic microbial communities are known to host among the most resistant and extreme-adapted organisms, our knowledge on microbial diversity and composition in this peculiar niche is still limited. In this study, we investigated the diversity and structure of the fungal assemblage in the cryptoendolithic communities inhabiting sandstone using a meta-barcoding approach targeting the fungal Internal Transcribed Sequence region 1 (ITS1). Samples were collected from 14 sites in the Victoria Land, along an altitudinal gradient ranging from 1,000 to 3,300 m a.s.l. and from 29 to 96 km distance to coast. Our study revealed a clear dominance of a ‘core’ group of fungal *taxa* consistently present across all the samples, mainly composed of lichen-forming and Dothideomycetous fungi. Pareto-Lorenz curves indicated a very high degree of specialization (F_0_ approximately 95%), suggesting these communities are highly adapted but have limited ability to recover after perturbations. Overall, both fungal community biodiversity and composition did not show any correlation with the considered abiotic parameters, potentially due to strong fluctuations of environmental conditions at local scales.

## Introduction

The Victoria Land region in Antarctica encompasses a latitudinal gradient of 8°, from the Darwin Glacier (79°S) in the South, to Cape Adare (71°S) in the North. Along with the widest area of the McMurdo Dry Valleys of the Southern Victoria Land, mountain tops and nunataks hanging from the polar plateau in the Northern Victoria Land are ice free-areas, and the exposed naked rocks represent the main substratum for microbial life (Nienow and Friedmann, 1993), hosting the highest standing biomass in this area (Cowan and Tow, 2004; Cary et al., 2010; Cowan et al., 2010; Archer et al., 2017). The dramatic temperature fluctuation (Nienow et al., 1988a; Nienow and Friedmann, 1993), extremely low relative humidity, and scarce liquid water availability in this area constrain terrestrial ecosystem processes, leaving the microbes as the only life forms able to persist in one of the most extreme environments on Earth (Nienow and Friedmann, 1993; Vincent, 2000; Zucconi et al., 2016). Given the harsh conditions related to osmotic, thermal and UV stresses throughout the interior of the Antarctic continent, microbial communities are forced to develop in cryptic habitats as a stress avoidance strategy (Pointing and Belnap, 2012). Except for the coastal sites, where the most permissive climatic conditions favor epilithic growth, life in the inland and high altitudes is predominantly present as endolithic colonization (Friedmann and Ocampo, 1976; Zucconi et al., 2016).

Studies focused on the isolation, identification, evolution and adaptation of microbial *taxa* from Antarctic rock communities revealed the occurrence of a surprising diversity of both prokaryotic and eukaryotic microorganisms, some of which are exclusive to this habitat (Selbmann et al., 2005, 2008, 2013, 2014a; Adams et al., 2006; Egidi et al., 2014). The taxonomic and functional diversity of bacteria from soil and hypolithic communities of the Miers Valley, in the McMurdo Dry Valleys of Antarctica, have been recently characterized (Wei et al., 2016), revealing a cyanobacteria-dominated community with a relatively high degree of functional redundancy; fungal soil communities are dominated by Chytridiomycota species (Dreesens et al., 2014). In contrast, the fungal endolithic diversity still remains largely unexplored (Zucconi et al., 2016; Archer et al., 2017). In particular, Zucconi et al. (2016) highlighted the importance of environmental parameters, mainly rock typology and to a lesser extent, altitude and distance to coast, in shaping microbial colonization of the lichen-dominated lithic communities, which are exceptionally widespread in the Victoria Land region. Cryptoendoliths prefer porous rocks and readily colonize sandstone. The structure of the fungal component of these communities has been investigated using a fingerprinting approach that revealed a high predominance of a few fungal species. This organization indicates a high degree of specialization of the community, with a consequent high resistance to stresses, but a poor resilience so that external perturbations may easily lead to possible extinctions (Selbmann et al., 2017).

The fungal diversity and *taxa* description of the Antarctic cryptoendolithic communities have been primarily investigated using culture-dependent approaches to identify dothideomycetous black yeasts, basidiomycetous yeasts and lichenized mycobionts (Selbmann et al., 2005, 2008; Egidi et al., 2014). With the development of culture-independent molecular methods, such as DNA meta-barcoding, a more accurate census of the microbial community is possible, resulting in a robust and comprehensive overview of the community composition both at global and local scales (Ji et al., 2013; Smith and Peay, 2014; Hiergeist et al., 2015; Tedersoo et al., 2015; Valentini et al., 2016). Commonly occurring organisms, that are shared among communities from the same habitat, are likely to play a crucial functional role in that particular assemblage (Shade and Handelsman, 2012), and the DNA meta-barcoding approach can be conveniently applied to have a better understanding of biodiversity and ecology of the ‘core’ fungal members.

Despite these recent advances, only rare studies on Antarctic endoliths have been carried out limited on a few rock samples or on different samples from a single location (de la Torre et al., 2003; Pointing et al., 2009; Archer et al., 2017). Moreover, these studies also have focused almost exclusively on the bacterial compartment (Wei et al., 2016).

In this study, we utilized an ITS meta-barcoding strategy to investigate the diversity, composition and distributional patterns of the fungal communities colonizing sandstone samples from 12 localities and 14 sites spanning from North to South Victoria Land, Antarctica. This research aimed to (i) explore the fungal diversity and community composition related to an altitudinal (m a.s.l.) and distance to coast (km) gradients, (ii) define the ‘core’ group of fungal *taxa* associated with such communities (iii) investigate the structure of such extreme-adapted communities. These data may give clues for improving the accuracy of predictions on the effects of climate change on polar microbial diversity and potentially aid in developing strategies to preserve the biodiversity of these unique ecosystems.

## Materials and Methods

### Study Area

**FIGURE 1:**
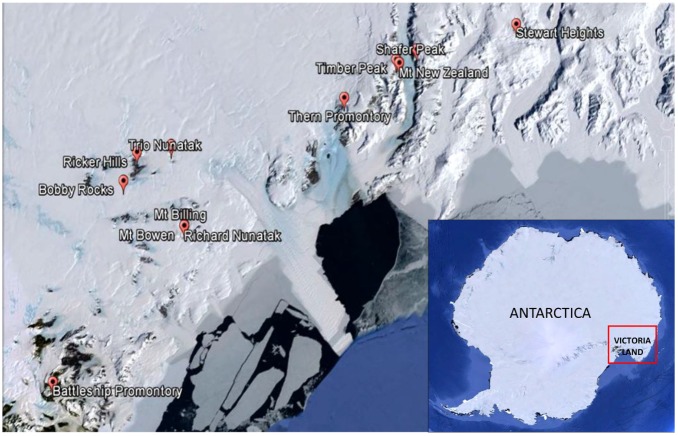
Map of the study area showing the location of the sampling sites. All locations visited were in Northern Victoria Land, except for Battleship Promontory, the only one visited in Southern Victoria Land. Victoria Land is pointed in the Antarctic continent.

**FIGURE 2:**
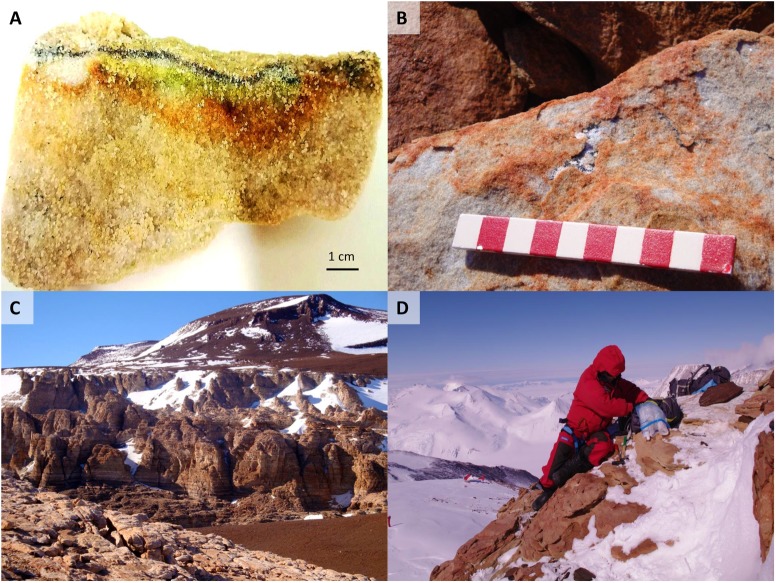
**(A)** Cryptoendolithic lichen dominated community colonizing a sandstone sample at Battleship Promontory, Southern Victoria Land. **(B)** A diffuse endolithic colonization on and within rock at Mt Billing. **(C)** Sandstone outcrops at Battleship Promontory. **(D)** Laura Selbmann sampling at Mt New Zealand, Northern Victoria Land, during the XXVI Italian Campaign (2010–2011).

Sandstone outcrops distributed along a latitudinal transect ranging from 73°29′26′′S (Stewart Heights, Northern Victoria Land) to 76°54′36′′S (Battleship Promontory, McMurdo Dry Valleys, Southern Victoria Land) from 1000 m a.s.l. (Battleship Promontory) to 3300 m a.s.l. (Shafer Peak site 2) and from 29 km (Thern Promontory) to 96 km (Ricker Hills) distance to coast (**Table 1** and **Figures 1**, **2**) were sampled. All sites were located in Northern Victoria Land, except for Battleship Promontory, located in Southern Victoria Land.

**Table 1:** List of sampling sites following an altitudinal gradient, with altitudes, distances to coast and geographic coordinates.

Locations	Altitude (m a.s.l.)	Distance to coast (km)	Coordinates
Battleship Promontory	1000	33.5	76°54′36′′S 160°56′05′′E
Trio Nunatak site 1	1000	82	75°30′02′′S 159°40′28′′E
Ricker Hills	1115	96	75°38′39′′S 159°01′42′′E
Mt Billing	1300	44	75°42′12′′S 160°54′28′′E
Trio Nunatak site 2	1400	84.5	75°28′59′′S 159°35′21′′E
Thern Promontory	1500	29	74°33′00′′S 162°04′00′′E
Bobby Rocks	1680	91	75°48′35′′S 159°11′15′′E
Mt Bowen	1874	39.5	75°45′24′′S 161°03′46′′E
Richard Nunatak	2000	71.6	75°56′53′′S 159°42′57′′E
Stewart Heights	2670	74	73°29′26′′S 163°54′44′′E
Timber Peak	2800	49.5	74°10′13′′S 162°25′31′′E
Mt New Zealand	2888	47	74°10′46′′S 162°31′01′′E
Shafer Peak site 1	3100	59	74°02′19′′S 162°37′16′′E
Shafer Peak site 2	3300	48	74°02′19′′S 162°37′16′′E

Samples were collected during the XXVI Italian Antarctic Expedition (2010–2011); the presence of lithic colonization was first assessed by direct observation *in situ* using a magnifying lens. Sandstone were excised using a geological hammer and sterile chisel, and samples placed in sterile bags, transported and stored at -20°C at the Tuscia University (Viterbo, Italy) until downstream analysis. The rocks investigated in this study are part of a larger set of different sandstone, granite, dolerite, quartz and lava-dike samples collected during the same expedition and analyzed using DGGE approach (Selbmann et al., 2017).

### DNA Extraction and Sequencing

Three rock samples from each site were crushed under sterile conditions by Grinder MM 400 RETSCH (Verder Scientific, Bologna, Italy) and 0.3 g were used for DNA extraction using MOBIO Power Soil DNA Extraction kit (MO BIO Laboratories, Carlsbad, CA, United States), according to the manufacturer’s protocol. The ITS1 region was amplified using ITS1F (CTTGGTCATTTAGAGGAAGTAA) and ITS2 (GCTGCGTTCTTCATCGATGC) primers developed for short read length (White et al., 1990; Smith and Peay, 2014). The PCR reactions were carried out in duplicate with a total volume of 25 μl, containing 1 μl of each primer, 12.5 μl of Taq DNA Polymerase (Thermo Fischer Scientific Inc., Waltham, MA, United States), 9.5 μl of nuclease-free water (Sigma–Aldrich, United Kingdom) and 5 ng of DNA. PCR conditions were: initial denaturation at 93°C for 3 min, 35 cycles of denaturation at 95°C for 45 s, annealing at 50°C for 1 min, extension at 72°C for 90 s, followed by a final extension at 72°C for 10 min in an automated thermal cycler (Bio-Rad, Hercules, CA, United States). The obtained amplicons were purified with Qiagen PCR CleanUp kit (Macherey-Nagel, Hoerdt, France) and normalized after quantification with the Qubit dsDNA HS Assay Kit (Life Technologies, United States). The equimolar pool of uniquely barcoded amplicons was paired-end sequenced (2 × 300 bp) on an Illumina MiSeq platform at the Institute for Integrative Genome Biology, University of California, Riverside.

Two replicates for each rock sample were sequenced and sequence reads from the sample replicates were merged to increase the amount of sequence information (Supplementary Table S1).

### Amplicon Sequencing Data

The ITS1 datasets were processed with the AMPtk: Amplicon ToolKit for NGS data (formally UFITS) v.0.9.3^1^ (Palmer et al., 2018). Barcodes and primers were removed from the amplicons sequencing data and reads were demultiplexed with split_libraries.py function in QIIME v 1.9.1 (Caporaso et al., 2010). Reads were subjected to quality trimming, PhiX screening, and chimera removal in AMPtk utilizing USEARCH with default parameters (v. 9.1.13) (Edgar, 2010). Singletons were removed and rare OTUs (<5 reads) were additionally trimmed off as recommended by Lindahl et al. (2013). The cleaned individual sample sequence files were merged into a single file clustered to identify molecular Operational Taxonomic Units (OTUs) with a 97% identity threshold using the VSEARCH (v 2.3.2) (Rognes et al., 2016) algorithm. Taxonomic identification was performed with SINTAX/UTAX (Edgar, 2010). Non-fungal sequences were excluded from the downstream analysis.

In addition, we mapped the relative abundances of compositional ‘core’ OTUs, defined as being present in at least 75% of the analyzed samples. The matrix display function in PRIMER-E software v7 (Ltd. Plymouth, United Kingdom) (Clarke and Gorley, 2015) was used to illustrate and compare the relative abundance of the community ‘core’ members on a heat-map using log-transformed taxonomic counts, while a UPGMA clustering method was implemented to reveal similarities in the community composition among the sampled sites, calculating Bray–Curtis index.

We examined whether the sampling effort was adequate to capture the fungal community richness by generating species rarefaction curves and species accumulation plots using the ‘rarecurve’ and ‘specaccum’ functions in the package ‘vegan’ (v. 2.3-4; Oksanen et al., 2013) in R 3.2.0 (R Development Core Team, 2015).

All raw sequence data have been submitted to the GenBank databases under BioProject accession number PRJNA379160.

### Biodiversity and Statistical Analysis

Biodiversity indices were estimated on averaged data using Primer-E v7 to investigate species richness and evenness of the fungal community. Following Morris et al. (2014), our analyses included (i) species richness (S), estimated as a count of the total number of species found in each sample, (ii) the Shannon index (H’), a phylotype-based approach constructed using OTU groupings (Shannon and Weaver, 1949; Ludwig, 1988), (iii) the Simpson’s index of Dominance (1-D), calculated to measure the probability that two individuals randomly selected from a sample will belong to the same species (or some category other than species) (Simpson, 1949), and (iv) the Pielou’s equitability index (J’) (Pielou, 1969). The non-parametric Spearman’s correlation coefficients were calculated and graphically represented to explore relationships between the biodiversity indices and sampled localities (Spearman, 1904).

The variability in species composition of the communities (β diversity) among the 14 sites was calculated (Whittaker, 1960; Anderson et al., 2006). The relationship between the considered environmental variables (altitude and distance to coast) and β diversity was tested by multiple linear regression on distance matrices (MRM) (Legendre et al., 2005; Lichstein, 2007; Ptacnik et al., 2010) implemented in the ‘ecodist’ package (Goslee and Urban, 2007) of R version 3.4.2. In this analysis, the environmental distance matrices between sampling sites were regressed against the species composition dissimilarity, to verify the effect of altitude (m a.s.l.) and distance to coast (km) on β diversity. Environmental distances were quantified by means of the Euclidean distance between each pair of sites, while the pattern in community similarity was calculated with the Jaccard index, starting from the species occurrence matrix. The robustness of results was estimated performing 1,000 permutations on the original dataset, and *P*-values for MRM models were obtained by comparing each observed regression coefficient with the distribution of 1,000 permuted values. All statistical tests were considered significant at *P* < 0.05.

To further show the evenness of these communities, Lorenz distribution curves were set up based on the meta-barcoding profiles, and a functional organization index was obtained (*F*_o_). In this study, the Lorenz curves were also evaluated based on the Pareto principle (Pareto, 1897). The theoretical perfect uniformity, represented by a line with a slope of 45° (*F*_o_ = 25%) means that all species in the community have the same number of individuals. *F*_o_ value of 45% indicates a community where few species are dominant; higher values represent a highly specialized community where a small number of species are dominant, while the vast majority are present at low abundance (Marzorati et al., 2008; Chen et al., 2015; Wang et al., 2015).

## Results

### Amplicon DNA Sequencing and OTU Abundance

The multiplexed files contained 3,720,171 sequence reads, resulting in 1,097,371 fungal ITS rRNA gene reads passing the quality filtering step. Samples averaged 78,383 reads, with a minimum of 18,013 to a maximum of 297,482 reads per sample (**Table 2**). Sequence reads were obtained from all samples, including those collected at highest elevations, such as Mt New Zealand (2888 m a.s.l.) and Shafer Peak site 1 (3100 m a.s.l.) in Northern Victoria Land.

**Table 2:** Diversity metrics for fungal ITS rRNA gene sequencing for each site.

Sites	Reads	S	H′	1-D	J′
Battleship Promontory	80426	93	1.92	0.75	0.39
Trio Nunatak site 1	18013	17	0.55	0.77	0.21
Ricker Hills	139839	111	2.4	0.64	0.44
Trio Nunatak site 2	75956	47	1.19	0.57	0.30
Mt Billing	68369	40	1.59	0.66	0.38
Thern Promontory	57090	42	1.49	0.69	0.36
Bobby Rocks	297482	55	1.56	0.69	0.33
Richard Nunatak	74913	70	1.89	0.85	0.42
Mt Bowen	54834	30	1.12	0.58	0.32
Stewart Heights	41391	67	2.47	0.84	0.55
Timber Peak	60549	51	1.30	0.69	0.30
Mt New Zealand	40160	66	1.42	0.59	0.32
Shafer Peak site 1	57635	48	0.77	0.75	0.29
Shafer Peak site 2	30714	42	1.62	0.69	0.30

*Analysis included: number of reads, species richness (S), Shannon index (H′), Simpson’s Index of Dominance (1-D), and Pielou’s equitability index (J′).*

Singletons and rare *taxa* (<5 reads) were removed (87 out of 362 OTUs total). Clustering of OTUs was performed at 97% identity threshold, resulting in a total of 275 OTUs. About 5% of the OTUs were non-fungal and excluded from downstream analysis. Rarefaction curves of total fungal OTUs per rock sample rarely reached a plateau (Supplementary Figure S1). However, the curve of the species accumulation plots approached saturation, suggesting that additional samples would have recovered very few additional OTUs (Supplementary Figure S2).

### Fungal Community Description

**FIGURE 3:**
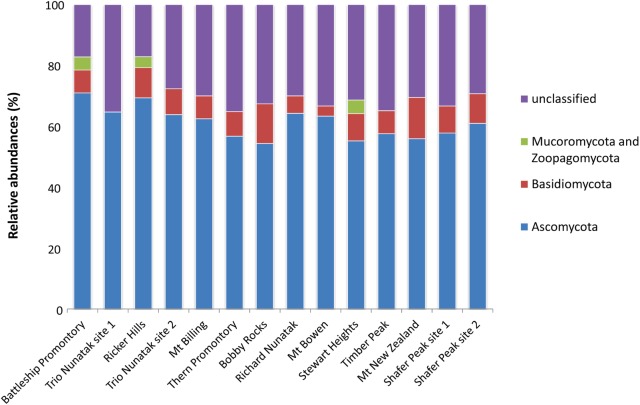
Relative abundances of the dominant fungal OTUs in the cryptoendolithic communities in Victoria Land, Antarctica. All relative abundances are based upon the sequences identified at the Phylum level.

About 25% of the total OTUs retrieved were unidentified at Phylum or sub-Phylum level. The majority of the identified fungal sequences recovered among all samples belonged to the Ascomycota (ranging from 55 to 70% of relative abundances), followed by Basidiomycota (from 4 to 12%), and Mucoromycota and Zoopagomycota (present at 5% only in three sites: Battleship Promontory, Ricker Hills, and Stewart Heights) (**Figure 3**). The relative abundance of Ascomycota and Basidiomycota ITS sequences varied among locations.

**FIGURE 4:**
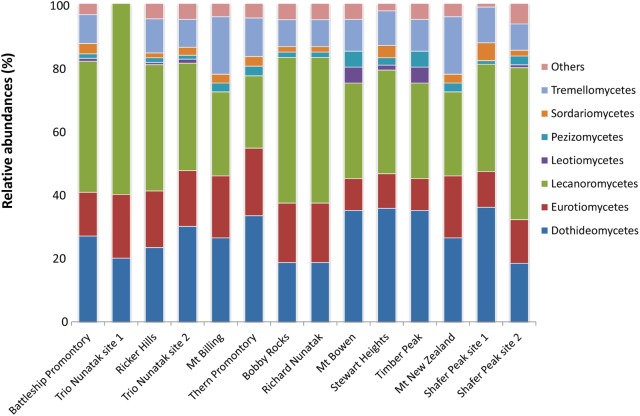
Relative abundances of the dominant fungal OTUs in the cryptoendolithic communities in Victoria Land, Antarctica. All relative abundances are based upon sequences identified at the class level. Others include classes Agaricomycetes, Microbotryomycetes, Saccharomycetes, Taphrinomycetes, and Cystobasidiomycetes, with relative abundances ≤ 5%.

At the class level, OTUs distribution varied among sites (**Figure 4**). Lichenized fungi in the Lecanoromycetes (Ascomycota) were the most abundant *taxa* and occurred in all analyzed samples (relative abundance ranging from 23 to 60%), followed by the ascomycetous classes Dothideomycetes (10 to 30%) and Eurotiomycetes (10–20%). The Tremellomycetes (Basidiomycota) were present at 10% of relative abundance in most of sites and totally absent in Trio Nunatak site 1; Agaricomycetes, Saccharomycetes, and Taphrinomycetes were the rarest members in these communities, detected only in few sites, mainly Thern Promontory, Mt Bowen, Stewart Heights, and Shafer Peak site 2.

The fungal ‘core’ community (i.e., OTUs present in at least 75% of the samples), was composed by 47 OTUs (**Table 3**). Fourteen OTUs were unclassified beyond the Kingdom level, while most OTU ‘core’ components belonged to the phylum Ascomycota and only three OTUs were assigned to the phylum Basidiomycota, including the former genus *Cryptococcus* sp. and *Solicoccozyma aeria.* Among the Ascomycota, most *taxa* belonged to the classes Lecanoromycetes and Dothideomycetes, followed by Sordariomycetes, Pezizomycetes, and Eurotiomycetes, which were present at lower percentage.

**Table 3:** Fungal ‘core’ composition (OTUs present in ≥75 samples) 97% of identity.

Taxonomic assignment		
OTU id	Phylum (confidence > 1)	Identification (confidence > 0.97)
OTU1	Ascomycota	*Buellia* sp.
OTU10	Ascomycota	*Friedmanniomyces endolithicus*
OTU104	Ascomycota	*Alternaria* sp.
OTU106	Ascomycota	Acarosporaceae
OTU109	Ascomycota	*Lecidea laboriosa*
OTU11	Basidiomycota	*Cryptococcus* sp.
OTU110	Ascomycota	Dothideales
OTU117	Unclassified	–
OTU118	Unclassified	–
OTU122	Ascomycota	Pezizales
OTU126	Ascomycota	–
OTU127	Unclassified	–
OTU13	Basidiomycota	–
OTU130	Unclassified	–
OTU133	Ascomycota	Dothideomycetes
OTU137	Unclassified	–
OTU14	Ascomycota	*Acarospora* sp.
OTU146	Ascomycota	*Sarcinomyces crustaceus*
OTU151	Ascomycota	*Verrucari*a sp.
OTU158	Ascomycota	*Acarospora* sp.
OTU161	Basidiomycota	*Solicoccozyma aeria*
OTU169	Unclassified	–
OTU17	Ascomycota	*Acarospora* sp.
OTU178	Ascomycota	Pleosporales
OTU18	Ascomycota	Acarosporaceae
OTU186	Unclassified	–
OTU119	Ascomycota	*Saitoella coloradoensis*
OTU199	Ascomycota	Sporormiaceae
OTU2	Ascomycota	*Lecidea cancriformis*
OTU203	Ascomycota	Pleosporales
OTU205	Ascomycota	Pleosporales
OTU207	Unclassified	–
OTU7	Ascomycota	*Cryomyces antarcticus*
OTU211	Ascomycota	Eurotiomycetes
OTU67	Ascomycota	*Aspergillus* sp.
OTU217	Unclassified	–
OTU23	Ascomycota	*Fusarium proliferatum*
OTU227	Unclassified	–
OTU26	Ascomycota	Sporormiaceae
OTU28	Unclassified	–
OTU281	Ascomycota	Acarosporaceae
OTU29	Ascomycota	*Aureobasidium pullulans*
OTU345	Unclassified	–
OTU353	Ascomycota	Parmeliaceae
OTU351	Ascomycota	Lecanorales
OTU37	Ascomycota	*Penicillium* sp.
OTU4	Ascomycota	Lecanorales

**FIGURE 5:**
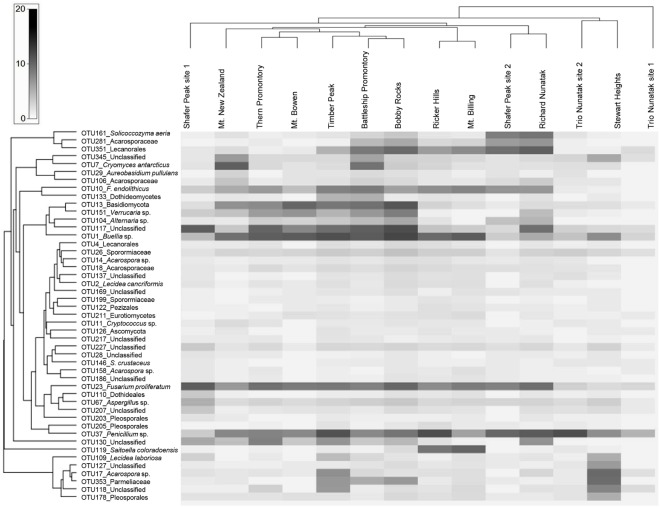
Heat map of the ‘core’ *taxa* relative frequency and UPGMA hierarchical clustering of samples. Values are scaled (log transformed) by *taxon* relative frequency across all samples. Frequencies are indicated by the color intensity: dark gray indicates a *taxon* with high relative frequency; white indicates absence and light gray represents low relative frequency. Both the ‘core’ OTUs and sites were clustered using a Bray–Curtis index.

We further examined the distribution of the fungal *taxa* from the ‘core’ community. Several *taxa* were present at all sites including *Solicoccozyma aeria* (OTU 161), unidentified Lecanorales (OTU 351), the unclassified *taxa* OTUs 345-117-227, one unidentified Acarosporaceae (OTU 281), *Cryomyces antarcticus* (OTU 7), *Friedmanniomyces endolithicus* (OTU 10), unidentified Basidiomycota (OTU 13), *Buellia* sp. (OTU 1), unidentified Sporormiaceae (OTU 26), *Fusarium proliferatum* (OTU 23) and *Penicillium* sp. (OTU 37). The distribution of *taxa* abundance was generally uniform across all the altitudes. Other ‘core’ *taxa*, such as unidentified Dothideomycetes (OTU 133), the unclassified *taxa* OTUs 137-217-186, unidentified Dothideales (OTU 110) and unidentified Pleosporales (OTUs 203–205), were present only intermittently among sites. Sampled sites were also hierarchically clustered by OTU abundance to examine patterns of similarities in community composition but did not exhibit any clustering by sampled localities (**Figure 5**).

### Diversity and Statistical Analysis

Species richness (S), Shannon (H′), Simpson (1-D), and Pielou (J′) indices for each site were calculated and reported in **Table 2**. The highest observed fungal richness (111 OTUs) was recorded at Ricker Hills (1400 m a.s.l.). In contrast, the Trio Nunatak site 1 (1000 m a.s.l.) exhibited low fungal richness, with only 17 OTUs retrieved. Battleship Promontory, Richard Nunatak and Stewart Heights showed the highest values for the other biodiversity indices. Among all sites, H′ ranged from 0.55 to 2.47, 1-D from 0.57 to 0.84 and J′ from 0.21 to 0.55.

**FIGURE 6:**
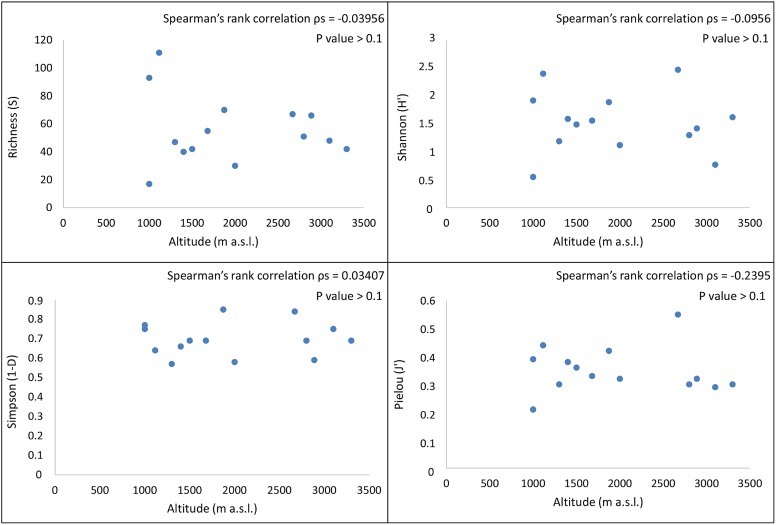
Spearman’s correlation coefficients between the biodiversity indices calculated on 14 endolithic communities and altitudinal gradient. A significant correlation between fungal diversity and altitude was not found when R, H′, D, and J′ indices were considered (Spearman’s correlation coefficient, *P*-value > 0.1).

Spearman’s ρ values represented the correlation between biodiversity indices and sampled sites. In all cases we found no significant correlation between diversity indices and locations, even among sites located at similar altitudes (*P*-values > 0.1) (**Figure 6**). A similar trend was between biodiversity and distance to coast (data not shown).

The variability in species composition of these communities (β diversity) across the 14 sites was measured with a Jaccard index, and the contribution of different geographic parameters (altitude: m a.s.l.; distance to coast: km) in shaping the fungal communities was estimated using an MRM analysis. The total β diversity varied among all locations. Highest similarity values occurred between Timber Peak (2800 m a.s.l., 49.5 km) and Bobby Rocks (1680 m a.s.l., 91 km) (80% of similarity) and between Timber Peak, Mt Billing (1300 m a.s.l., 44 km) and Ricker Hills (1115 m a.s.l., 96 km) (70% of similarity). The highest values of dissimilarity were obtained between Mt Bowen (1874 m a.s.l., 39.5) and Battleship Promontory (1000 m a.s.l., 33.5) (only 30% of similarity). Even sites from the same locations (i.e., Trio Nunatak site 1 and 2, Shafer Peak site 1 and 2) showed low percentages of similarity. In addition, MRM analysis indicated no correlation between the fungal community composition and either altitude (m a.s.l.) or distance to coast (km) (data not shown) among all sites (*P*-value > 0.05).

### Pareto Lorenz Curves

**FIGURE 7:**
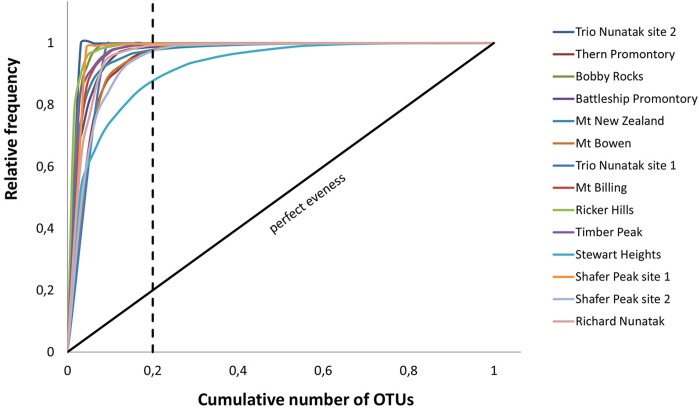
Pareto-Lorenz curves. The analysis was based on number of OTUs and their frequencies in the meta-barcoding profiles. Theoretical perfect evenness (perfect evenness line) is characterized by a curve close to the 45°C diagonal.

Pareto-Lorenz curves distribution patterns of the meta-barcoding profiles were plotted based on the numbers of OTUs and their frequencies. *F*_o_ values were high in all sampled sites, ranging from 86 to 100%. These results indicate these fungal endolithic communities were dominated by very few, abundant, and specialized species and some other rare species (**Figure 7**).

## Discussion

Endolithic microbial communities occur globally and play an important role in biogeochemical processes, including rock and mineral transformations, bio-weathering, and biomineral formation (Gadd et al., 2012), particularly in border ecosystems (Nienow and Friedmann, 1993). Nevertheless, little is known about the composition, diversity and distribution of these communities, especially in continental Antarctica, where they represent the predominant life form (Nienow and Friedmann, 1993). Sequence-based fungal communities have been retrieved from samples in all locations, including the highest altitude sites, such as Shafer Peak (3300 m a.s.l.) and Mt New Zealand (3100 m a.s.l.) in Northern Victoria Land, previously reported as colonized by fungal populations (Zucconi et al., 2016).

Most fungi in the Antarctic mycobiome are Ascomycetes. This is supported by the predominance of ascomycetous OTUs in our study and previous molecular surveys of soil Antarctic communities (Cox et al., 2016; Ji et al., 2016; Wei et al., 2016), chasmoendolithic communities in Miers Valley, McMurdo Dry Valleys (Yung et al., 2014), and in association with mosses in ice-free coastal outcrops (Hirose et al., 2016). The majority of the Ascomycota were identified as members of the lichen-forming class Lecanoromycetes, followed by Dothideomycetes, the widest and most diverse class in the Ascomycota. Our metabarcoding observations are consistent with 20 years of culture-based isolation and identification, where the Dothideomycetes were the most frequently isolated fungi from these communities (Selbmann et al., 2005, 2008; Egidi et al., 2014). In contrast, Lecanoromycetes are infrequently detected with standard isolation procedures, likely due to difficulties in culturing these fungi with obligate symbiotic lifestyles. However, the widespread presence of Lecanoromycetes detected with molecular approaches is not surprising, as lichen-forming fungi have been reported as dominant in cryptoendolithic communities colonizing sandstone rocks in Victoria Land (Friedmann et al., 1988; Cockell et al., 2003; Zucconi et al., 2016). Similarly, lichens predominate in many other continental Antarctic localities, where cold-adapted mycobionts have been previously recorded (e.g., Ruprecht et al., 2010, 2012). In this study, *Lecidea* sp. (family Lecideaceae; class Lecanoromycetes) and *Buellia* sp. (family Physciaceae; class Lecanoromycetes) were recorded in almost all the analyzed samples. The genus *Buellia* encompasses species considered endemic to Antarctica, such as *Buellia frigida*, a crustose lichen which grows on rock surfaces in ice-free areas of Antarctica and has been widely found in both coastal and mountain locations across the continent (Jones et al., 2015). Similarly, *Lecidea* species live endolithically in granite rocks of continental Antarctica (de Los Rìos et al., 2005). Lichens are considered exceptionally well adapted to the lithic lifestyle, thanks to their low mineral nutrient demand, high freezing tolerance, and ability to be photosynthetically active at suboptimal temperatures (Kappen, 2000). Consistent with our findings, a recent study on lithic colonization patterns from an additional locality in the McMurdo Dry Valleys, University Valley, Southern Victoria Land, documented a lichen mycobiont prevalence in sandstone communities (Archer et al., 2017).

In addition to the lichen-forming *taxa*, *Friedmanniomyces endolithicus* (order Capnodiales; class Dothideomycetes) is a ‘core’ member of the sandstone cryptoendolithic community as it was found in the majority of the analyzed samples, confirming the widespread presence of *Friedmanniomyces* spp. in Northern Victoria Land. The genus *Friedmanniomyces* includes two described species of rock-inhabiting meristematic black fungi, *F. simplex* and *F. endolithicus* (Onofri et al., 1999; Selbmann et al., 2005). The genus is endemic to Victoria Land and the most-frequently isolated non-lichenized fungus from rock substrata in this area (Selbmann et al., 2005, 2015; Ruisi et al., 2007). *Friedmanniomyces* spp., a well-studied genus from the phylogenetic, taxonomic, and physiological perspective (Selbmann et al., 2005; Egidi et al., 2014), possess stress-tolerant adaptations which allow them to inhabit inert surfaces and survive long time under dry conditions (Onofri et al., 2004). We also identified *Cryomyces antarcticus* (class Dothideomycetes) as a community ‘core’ member, although occurring at a relatively lower abundance compared to the other dominant *taxa*. The genus *Cryomyces* encompasses four species of cold-adapted rock-inhabiting black yeasts; *Cryo. montanus* and *Cryo. funiculosus* have been isolated from Alpine rocks collected above 3000 m a.s.l (Selbmann et al., 2014a), while the two Antarctic species *Cryo. antarcticus* and *Cryo. minteri* have been isolated primarily from the McMurdo Dry Valleys in Southern Victoria Land (Selbmann et al., 2005), and rarely in Northern Victoria Land (Cecchini, 2015). Our results suggest a wider distribution for *Cryo. antarcticus* in the continent than previously recorded, highlighting the higher power of meta-barcoding approaches compared to the culture-dependent approach in recovering low abundant, even if cultivable, slow growing fungi.

Additional members of the Ascomycota were found in the cryptoendolithic fungal communities, including one *Aspergillus* sp. (family Trichocomaceae; class Eurotiomycetes) and several unidentified *taxa* belonging to the order Pleosporales (Dothideomycetes). Several members of the Pleosporales have been previously associated with rock formations in both cold and hot areas (Ruibal et al., 2009; Egidi et al., 2014). Filamentous fungi such as *Aspergillus* spp. in Antarctica have been isolated and described from oligotrophic soils (Godinho et al., 2015), but never from rocks. Indeed, the environmental pressure typically associated with the exposed polar rocks requires a high degree of specialization (Selbmann et al., 2005, 2013; Onofri et al., 2007), suggesting this substratum is not suitable for fast-growing, cosmopolitan *taxa*, such as members of the genus *Aspergillus*. Therefore, although a long-range aerial spore dispersal cannot be completely excluded (see Pearce et al., 2009), we hypothesize that the occurrence of *Aspergillus* spp. in our dataset is more likely the result of a post-sampling contamination, rather than the reflection of a true component of the Antarctic rock mycobiome. Basidiomycota represents a rare fraction of the ‘core’ community. Only three Basidiomycota phylotypes were recovered, two identified as *Cryptococcus* sp. and one to the taxonomically related species *Solicoccozyma aeria* (former *C. aerius*) as recurring community members. Although members of the former basidiomycetous genus *Cryptococcus* have been isolated globally, including Antarctica, both from rock and soil communities (Vishniac, 1985; Vishniac and Kurtzman, 1992; Montes et al., 1999; Scorzetti et al., 2000), the recent taxonomic revision of the Tremellomycetes (Liu et al., 2015) positioned this polyphyletic genus into many new *taxa*, thus modifying the taxonomical and ecological picture of the yeast distribution in polar and subpolar ecosystems. Accordingly, species of the genera *Goffeauzyma*, *Naganishia*, *Papiliotrema*, *Solicoccozyma*, *Vishniacozyma*, and *Phenoliferia* have been frequently found in both polar and non-polar cold ecosystems (Buzzini et al., 2017; Sannino et al., 2017). Although their association with rock substrates from cold sites worldwide has been previously reported by Selbmann et al. (2014b), overall Basidiomycota are infrequently found in cryptoendolithic community, making up at most 3% or less of fungal sequences on the sub-Antarctic, low maritime and high maritime Antarctic (Cox et al., 2016). New species from class Taphrinomycetes have been repeatedly isolated and described as endemic Antarctic species (Selbmann et al., 2014c), but were rarely identified in this dataset. Interestingly, we have been able to retrieve members of Saccharomycetes at low abundance, although these *taxa* have never been isolated from Antarctic cryptoendolithic communities. The overall low biodiversity indices values obtained in this study are consistent with recent observations of Antarctic lithic communities in Victoria Land on different rock typology (Selbmann et al., 2017). Compared with species richness indices of soil microbial communities of Antarctic Peninsula our endolithic mycobiome diversity is very low (Chong et al., 2009), indicating a strict predominance of a restricted number of specialized species. The fungal community composition did not appear influenced by elevation: indeed, samples collected on the top of Mt New Zealand (2888 m a.s.l.), Stewart Heights (2670 m a.s.l.), and Richard Nunatak (2000 m a.s.l.), had biodiversity richness values similar to the ones detected at lower altitude, e.g., Trio Nunatak (1400 m a.s.l.), Ricker Hills (1115 m a.s.l.), Battleship Promontory (1000 m a.s.l.), and Mt Billing (1300 m a.s.l.).

There is a remarkable variability in the occurrence of lichen-forming and meristematic fungi, across altitudes, suggesting that this parameter alone is not an important driving factor of fungal community composition. The black yeast *Cryo. antarcticus* was consistently present along all localities, suggesting that this ‘core’ *taxon* is remarkably resistant to ecological stresses. The members of the *Cryomyces* genus are among the most extreme environment resistant species know to date: they are able to resist extreme temperatures (-20 up to 90°C) and UV radiation (Onofri et al., 2007; Selbmann et al., 2011; Pacelli et al., 2017). *Cryo. antarcticus* strains have withstood ground simulated space and Mars conditions (Pacelli et al., 2017) and 18 months of real Space exposure and Mars-simulated exposure outside the International Space Station (Onofri et al., 2012, 2015, 2018). Based on this resilience, *Cryo. antarcticus* is considered among the best eukaryotic models for astrobiological studies.

The functionality (*F*_o_) of these communities, represented by the Pareto-Lorenz curves, was mostly around 100% (**Figure 7**). The theoretical perfect uniformity (e.g., a slope of 45°, *F*_o_ = 25%) means all species in the community have the same number of individuals. Values of *F*_o_ near 100% indicate a highly specialized community where few species dominate, while all others are present as few representatives (Marzorati et al., 2008). Our results indicate that all the examined communities have a high degree of specialization and adaptation, but lack resilience to external perturbations.

An exacerbated level of specialization in such communities also implies a lack resilience to external perturbations, as reported by Selbmann et al. (2017).

The Spearman’s coefficient correlation confirms that fungal biodiversity (i.e., number of OTUs retrieved and biodiversity indices) was not related to the sampled sites and environmental parameters considered in this study (i.e., altitude and distance to coast). These data were further supported by MRM analysis, which did not show any correlation between environmental variables and community composition, highlighting also a high level of dissimilarity in samples collected in same locations. These results lead to the conclusions that the environmental variables here considered (altitude and distance to coast) did not play a role in shaping the fungal community diversity and composition in these peculiar ecosystems. Selbmann et al. (2017) found a slight negative effect of these of these two parameters on fungal biodiversity in a study based on a much more heterogeneous sampling.

Nevertheless, it can be expected that in border ecosystems, particularly susceptible to environmental changes (Parmesan, 2006), even minimal local variations in environmental conditions could be crucial for microbial life. Therefore, additional parameters as water availability, rock temperature and sun exposition, might be considered in the future. For this reason, sensors for a constant monitoring of these parameters have already been installed in some sites of both northern and southern Victoria Land. Combined data on the climatic and environmental conditions, and their daily and seasonal variation, will be of help to elucidate the processes influencing biodiversity variations, community composition and species extinction. These parameters, monitored in the long run, may allow to predict possible consequences of Climate Change on terrestrial biota in Antarctica (Friedmann et al., 1987; Nienow et al., 1988; McKay et al., 1993).

This study is the largest attempt to comprehensively identify patterns of fungal diversity in rocks in Antarctica along a gradient of altitude and distance to coast. With the advent of NGS technologies, our understanding of Antarctic microbial diversity and evolution is improving, but further investigation is required to elucidate how microbiota responds to environmental pressure and, in the long run, how future environmental changes will impact these unique communities (Tilman, 1996; Van Horn et al., 2014). The high degree of specialization and the low taxonomic richness found in this study alert on the potential high susceptibility of Antarctic endolithic communities to environmental changes (Tilman, 1996; Nielsen et al., 2012; Van Horn et al., 2014). Data obtained in this study are of importance to set a proper experimental plan in the future, taking into consideration additional environmental parameters and organizing a more targeted sampling aimed to provide a better evaluation of the potential consequences of environmental changes on this unique ecosystem.

## Author Contributions

Samples were collected by LS and LZ during the XXVI Italian Antarctic Expedition (2010–2011). CC performed the DNA extraction. CC and NP performed the PCR and sequencing preparation. CC, JS, and NP performed the data processing and analyses. CC, LS, LZ, and JS wrote the paper with input from SO, EE, NP, PB, and AF.

## Conflict of Interest Statement

The authors declare that the research was conducted in the absence of any commercial or financial relationships that could be construed as a potential conflict of interest.

## Footnotes

1 https://github.com/nextgenusfs/amptk
