# Supplementary material for: Antarctic Cryptoendolithic Fungal Communities Are Highly Adapted and Dominated by Lecanoromycetes and Dothideomycetes
Source: Front Microbiol. 2018 Jun 29;9:1392. doi: 10.3389/fmicb.2018.01392 (PMC6033990; doi:10.3389/fmicb.2018.01392)
Supplement: Supplementary file 1 [file Table_1.PDF]

Table 1S Average number of reads per replicate

Sites	Replicate 1	Replicate 2
Battleship Promontory	77854	82997
Trio Nunatak site 1	12271	10813
Ricker Hills	119767	159911
Trio Nunatak site 2	89123	62789
Mt Billing	68194	68544
Thern Promontory	66100	48080
Bobby Rocks	249217	345747
Richard Nunatak	81112	68714
Mt Bowen	54791	54877
Stewart Heights	41005	41777
Timber Peak	68783	52315
Mt New Zealand	44892	35428
Shafer Peak site 1	59440	55830
Shafer Peak site 2	37723	23705
