# Supplementary material for: Antarctic Cryptoendolithic Fungal Communities Are Highly Adapted and Dominated by Lecanoromycetes and Dothideomycetes
Source: Front Microbiol. 2018 Jun 29;9:1392. doi: 10.3389/fmicb.2018.01392 (PMC6033990; doi:10.3389/fmicb.2018.01392)
Supplement: Supplementary file 2 [file Image_1.PDF]

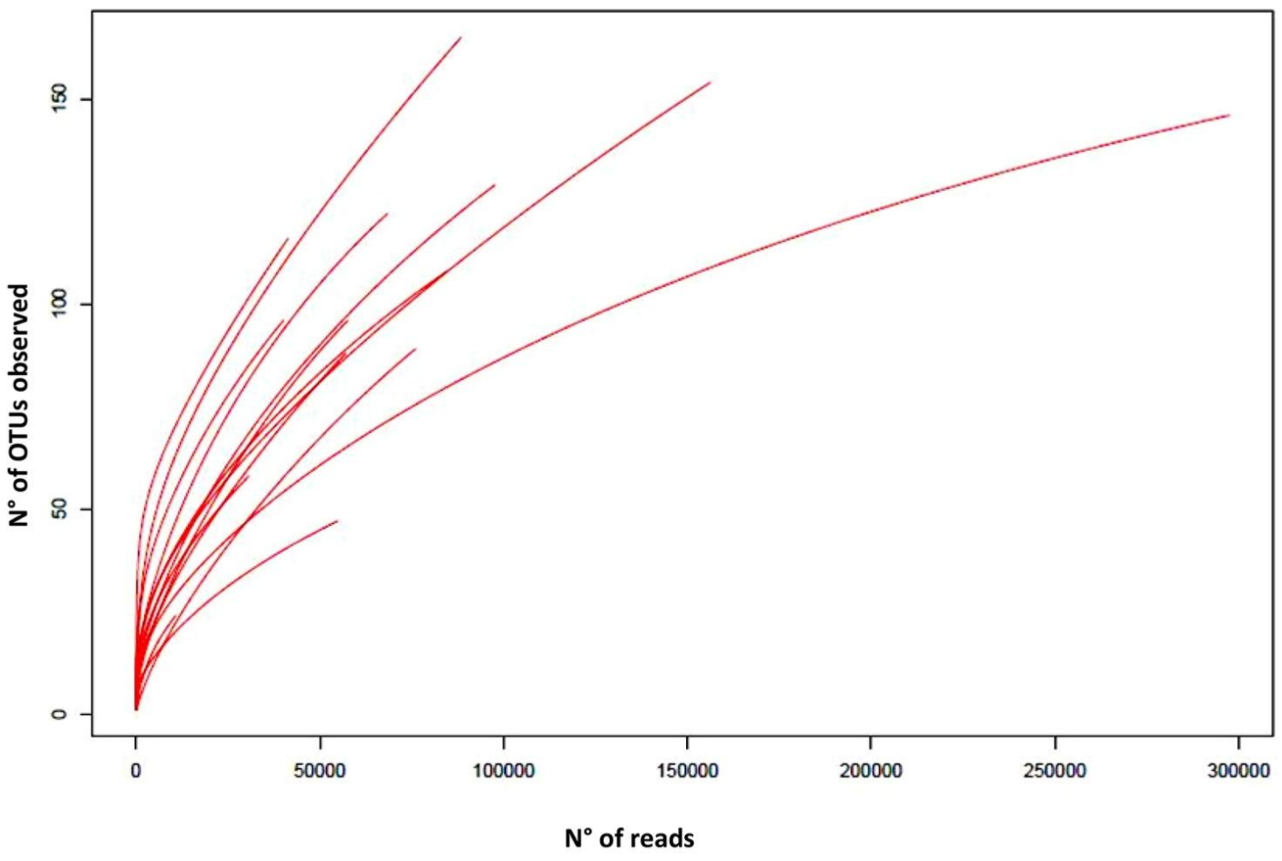

Figure 1S. The frequency of observed OTUs for each site was used to calculate fungal rarefaction curves using the ‘rarecurve’ function in the R library vegan.
