# Supplementary material for: Antarctic Cryptoendolithic Fungal Communities Are Highly Adapted and Dominated by Lecanoromycetes and Dothideomycetes
Source: Front Microbiol. 2018 Jun 29;9:1392. doi: 10.3389/fmicb.2018.01392 (PMC6033990; doi:10.3389/fmicb.2018.01392)
Supplement: Supplementary file 3 [file Image_2.PDF]

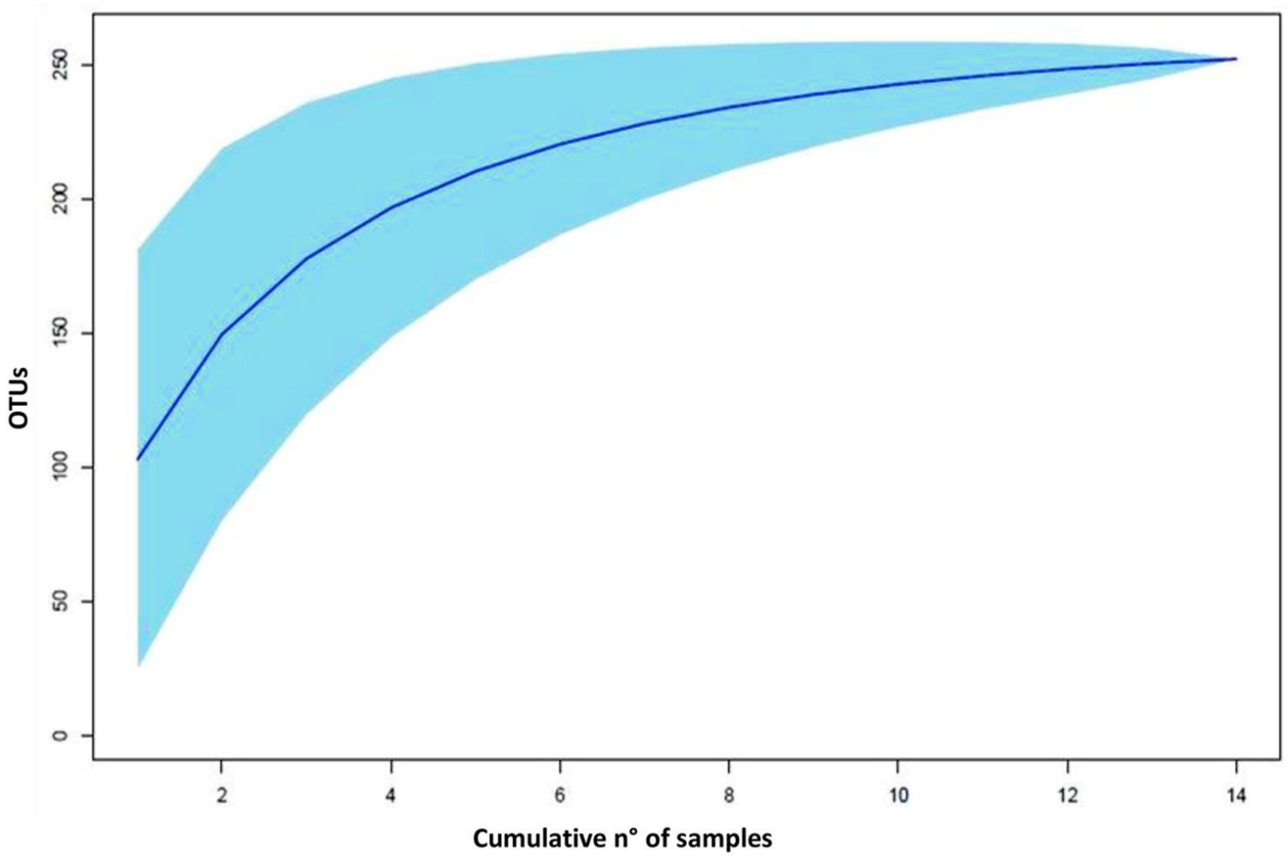

Figure 2S. Species accumulation curve, calculated using ‘specaccum’ function implemented in the R library vegan, represents the number of OTUs found at increasing number of samples.
Shaded area corresponds to the deviation standard.
